# Nurses’ experiences and sense making of COVID-19 redeployment and the impact on well-being, performance, and turnover intentions: A longitudinal multimethod study.

**DOI:** 10.1016/j.ijnsa.2024.100244

**Published:** 2024-09-21

**Authors:** Alice Dunning, Hannah Hartley, Kerrie Unsworth, Ruth Simms-Ellis, Michael Dunn, Angela Grange, Jenni Murray, Jayne Marran, Rebecca Lawton

**Affiliations:** a Sheffield Centre for Health and Related Research (SCHARR), Division of Population Health, University of Sheffield, Sheffield, S1 4DA UK; b Yorkshire Quality and Safety Research Group, Bradford Institute for Health Research, Temple Bank House, Bradford Royal Infirmary, Bradford, BD9 6RJ, UK; c Leeds University Business School, University of Leeds, Leeds, LS6 1AN, UK; d School of Psychology, University of Leeds, Leeds, LS2 9JT, UK; e Centre for Biomedical Ethics, Yong Loo Lin School of Medicine, National University of Singapore, 117597, Singapore

**Keywords:** Covid-19, Nurse redeployment, nurse wellbeing, nurse staffing, Sensemaking

## Abstract

**Background:**

During Covid-19 nurses were redeployed to new teams and specialties at a level never previously experienced. Little is known about how nurses made sense of and coped with this situation and what we can learn from this for future redeployment approaches.

**Objectives:**

We sought to understand how nurses made sense of ongoing redeployment during the COVID-19 pandemic and how this related to their psychological distress, burnout, turnover intentions, and perceived performance

**Design:**

A longitudinal multi-method design. (ISRCTN: 18,172,749).

**Setting(s):**

Three acute National Health Service (NHS) Trusts in England, selected for diversity in geographical location and ethnicity, with different COVID-19 contexts.

**Participants:**

Sixty-two nurses (90 % female; 83 % white) who experienced different types of redeployment during the pandemic, with an average of 17 year's post-registration experience (mean age 41 years).

**Methods:**

We gathered both interview and survey data from 62 nurses across two or three time points in 2020–2021 and sought to find commonalities and differences in patterns of experience using Pen Portrait analysis.

**Results:**

The pandemic redeployment process was life-changing for all nurses, personally and professionally**.** The research uncovered an intertwined pattern of identity and sensemaking as nurses coped with COVID-19 redeployment. Three sensemaking ‘journeys’ were evident, involving professional identity as a nurse and identification with one's organisation. Nurses in journey one: ‘Organisational Identification and Professional Identity Maintained’ (n = 28) had the best outcomes for wellbeing, burnout, performance, and retention. Those experiencing the ‘Devaluation of Organisational Identification But Maintenance of Professional Identity’ journey (n = 24) maintained their professional identity, but their organisational identification deteriorated. Journey three nurses: ‘Devaluation of both Organisational Identification and Professional Identity’ (n = 10) had the worst outcomes for wellbeing, burnout, performance, and retention. A salient nurse identity triggered stoicism and resilient behaviours while external cues of control, support and contextual awareness affected organisational identification.

**Conclusions:**

Nurses made sense of their experiences of redeployment during Covid-19 differently which, in turn affected their outcomes. Given the stark differences in how nurses perceived their psychological distress, burnout, turnover intentions and performance across the journeys, the importance of understanding the cues (e.g. having autonomy) associated with each journey is apparent. Thus, our research provides clear guidance for managers to help them support nurses during redeployment.

**What is already known about this topic**

Redeployment was key in the NHS response to managing the demands in the pandemic.

Working through COVID-19 had a huge impact on healthcare professionals’ wellbeing and burnout.

Problem-focused coping is more effective than emotion-focused coping when dealing with stressful situations, but ongoing extreme events like COVID-19 redeployment do not allow problem-focused coping to occur.

Alt-text: Unlabelled box

**What this paper adds**

We identified three sensemaking journeys associated with differing identity lenses (1. organisational identification and professional identity maintained; 2. professional identity maintained but organisational identification devalued; 3. devaluation of both organisational identification and professional identity). These show how nurses made sense of redeployment and how it impacted their identity as a nurse and their organisation identity.

These journeys were created through different experiences of control, support and contextual awareness and were associated with different outcomes for the nurses’ psychological distress, burnout, turnover intentions, and performance. The cues identified in the journeys demonstrate key aspects in redeployment which managers should address to support nurses in future redeployments.

Alt-text: Unlabelled box

## 1 Introduction

The COVID-19 pandemic imposed unprecedented demands on health services (Maben and Bridges, 2020) and, as part of the response, nurses were redeployed to support patient care Dunn et al. (2020). Redeployment – reassigning employees to new places or functions – is used to manage staffing numbers and patient safety by covering areas where the number and acuity of patients requires a greater number of staff Donnelly (2014); Saville et al. (2019). Mass redeployment was utilised throughout the pandemic - nurses were redeployed into unfamiliar settings, to high-risk areas or needed to change roles to allow shielding (Dunn et al., 2020) - and the situation was obviously a long-term, extreme event with significant and harmful effects Chatzittofis et al. (2021); Maben et al. (2022); Perry et al. (2018).

As the largest occupational group in healthcare with the most contact time with patients, nurses were particularly at risk during the pandemic Perry et al. (2018). Growing evidence indicates that nurse wellbeing, performance, and intentions to stay in nursing have all been adversely affected. For example, nurses were significantly more likely than doctors to suffer from depression and Post Traumatic Stress Disorder Chatzittofis et al., (2021). Systematic reviews have identified a related increase in nurse burnout since the pandemic (Galanis et al. (2021); Zareei et al. (2022). Redeployed into unfamiliar disciplines and teams while managing unprecedented demand for patient care (Dunn et al., 2020), nurses consistently describe experiencing moral distress and compassion fatigue during this time (Maben et al., 2022), unable to perform the nursing role they were trained for. The dilution of skill mix and fragmentation of patient care, according to intensive care nurses in one study, led to an increase in adverse events and poorer quality of care Stayt at al. (2022). Other studies highlight factors such as Personal Protective Equipment compromising ability to function (e.g. extreme discomfort, poor vision, communication issues) and extreme anxiety which impaired adaptation and decision making (e.g. Winanda et al., 2022). We have also witnessed a significant increase in nurses’ intention to leave their profession following the COVID-19 pandemic (Falatah, 2021; Maben et al., 2022). The way in which nurses’ experiences of redeployment during this prolonged crisis were related to changes in wellbeing, performance and turnover intentions remains less clearly understood and defined.

Sensemaking is a cognitive process that allows people to understand confusing, ambiguous, or equivocal events and situations Weick (1995). It is an ongoing process that is triggered and influenced by cues in the environment and social interactions Maitlis and Christianson (2014); Weick (1995). Our behaviours and actions usually occur without deliberative sensemaking Sandberg and Tsoukas (2015). However, when normality is disrupted, we look back over how we (and others) have behaved and generate a “story” that enables us to analyse and explain it Brown et al. (2008); Weick (1995). Although sensemaking and coping research tends to occur in different fields, there is growing recognition that emotions (emotion-focused coping) can disrupt a sense of normality, triggering sensemaking (Maitlis et al., 2013) and that sensemaking can then be used as a way of coping with negative emotions Dwyer et al. (2021). It can be argued that, for nurses, redeployment during the COVID-19 pandemic represented a disruption of normality which may have inhibited problem-focused coping and necessitated personal sense-making and a reliance upon emotion-focused coping.

Most sensemaking research has focused either on short-term crises such as bushfires (e.g., Weick, 1993) or longer-term events that are not life-threatening, such as organisational change (e.g., Bartunek et al., 2006; Gioia and Thomas, 1996). Sensemaking during, or following, short-term extreme contexts can affect one's view of one's role and required behaviours (e.g., Weick, 1993) and is influenced by environmental demands (e.g., Jensen and Mahmud, 2023) and social framing of the situation (e.g., Baran and Scott, 2010). This suggests that the way in which nurses made sense of the redeployment disruption during the COVID-19 pandemic may have affected not only their performance behaviours but also their well-being, burnout and perceptions of whether or not they wish to stay in their nursing job.

Unfortunately, we know little about how people make sense of, and emotionally cope with, a long-term, ongoing crisis. Christianson and Barton (2021) stated that COVID-19 has highlighted the need for research into extended periods of sensemaking. Concurrently, while research has begun to examine long-term sensemaking (e.g., Medeiros, et al., 2022; Stephens et al., 2020) this has not considered extreme events such as the “frontline” workplace of nurses during this time. Therefore, understanding nurse sensemaking and coping during long-term disrupted redeployment is important as nurses may face similar pandemics in the future and redeployment is ongoing due to workforce shortages, and hospital funding gaps.

In this study, we address the question: What were nurses’ experiences of redeployment during the COVID-19 pandemic and how did these impact upon well-being, performance and turnover intentions? Specifically, we examine how nurses made sense of redeployment over a prolonged period during the COVID-19 pandemic and their coping strategies. We focused on the effects of sensemaking on psychological distress, burnout, turnover intentions, and perceived performance. Identifying the cues and environmental demands that influenced these sensemaking narratives will extend our theoretical knowledge of sensemaking and have important practical implications for nurse managers.

## 2 Method

### 2.1 Study design

This study was part of a multi-stage longitudinal mixed-methods research programme exploring nurses’ experiences of redeployment during the pandemic. Qualitative and quantitative data were collected simultaneously in a convergent parallel mixed methods design Creswell (2014). Data were collected from March 2021 to March 2022, at three time-points: interviews were conducted at time-point one (T1) and three (T3), and online surveys at all three time-points with a one-to-three months gap between each survey. Mixed methods were used to both triangulate and elaborate upon findings (Gibson, 2017): Interviews provided the experiential information while surveys provided longitudinal patterns. Timings varied slightly between research sites due to COVID-19 waves causing staggered recruitment. Median collection points were July 2021 (T1), October 2021 (T2) and January 2022 (T3).

### 2.2 Participants and setting

Three NHS acute hospital Trusts in England were purposively sampled for workforce ethnic diversity and geographical locations. These three Trusts also experienced different patient need within the pandemic, for example one Trust had clear ‘waves’, and another experienced a sustained high level of patient demand. Nurses were contacted by nurse managers participating in the wider research project (Hartley et al. (2024) and recruited using purposive snowball sampling. Further targeted recruitment took place to ensure breadth of experience (i.e., redeployment to different settings, nurses from different bands or ethnic backgrounds), through sharing study information on mailing lists, forums, and social media posts (Twitter). A member of the research team also ‘walked the wards’ on sites to discuss the study with potential participants and hand out flyers.

### 2.3 Measurement tools and data collection

The work was inductive and used a subjectivist epistemology. As such, both qualitative and quantitative data were perceived to be contextualised rather than objective. After consent, participants were emailed a link to the online survey hosted on Qualtrics and invited to the first interview which took place virtually. Semi-structured interviews were conducted by two members of the research team (AD & HH). The same interviewer conducted T1 and T3 interviews to be able to follow up themes raised in the first interview. The interview schedule involved questions, seeking participants’ experiences and perception of their: pre and pandemic redeployment, impact on their mental health and patient care, how their organisation managed redeployment, and how they felt about their future as a nurse. Each interview lasted on average 62 minutes (range: 37- 95 minutes)

A 58-item survey instrument was created to capture experiences of outcomes. We used scales that had been previously validated, namely: The Oldenburg Burnout Inventory (Demerouti and Bakker, 2008) for burnout (disengagement and exhaustion subscales); General Health Questionnaire-12 (Goldberg, 1972; Hardy et al., 1999) for psychological distress; Nurse Performance scale (Greenslade and Jimmieson, 2007) for performance – specifically the subscales task-based performance [information, coordination of care, technical care] and support-based performance [social support, interpersonal support, job-task support]; and turnover intention items from Kim et al., (2020) (see Table 1). The same instrument was used at each time-point, except at T1 when additional demographic questions (including age, sex, and ethnicity) were included.

**Table 1:** Outcomes and measures used in the surveys.

Outcome	Pre-Validated Measure	Number of items	Scoring	Reliability
Burnout	Oldenburg Burnout Inventory (Demerouti and Bakker, 2008) Subscales: disengagement and exhaustion	16	Range 1–5 High, moderate, low burnout calculated by splitting data into thirds Low disengagement/exhaustion (scores<2.44); moderate disengagement/exhaustion (scores 2.44–3.31); high disengagement/exhaustion (scores>3.31)	Cronbach alphas, Disengagement: T1 0.66, T2 0.78, T3 0.84 Exhaustion: T1 0.81, T2 0.86, T3 0.92
Psychological distress	General Health Questionnaire-12 (GHQ-12) (Goldberg, 1972; Hardy et al.,)	12	Range 1–4 Typical distress (score<19), evidence of distress (score 20–27), severe psychological distress (score >28)	Cronbach alphas: T1 0.83, T2 0.92, T3 0.90
Job performance	Nurse Performance Scale (Greenslade and Jimmieson, 2007) Subscales: task- based and support based	27 (merged & omitted items irrelevant to COVID context)	Range 1–7 Below expectations (scores 1–2), meets expectations (scores 3–5), exceeds expectations (6–7)	Cronbach alphas, Task-based: T1 0.90, T2 0.96, T3 0.94 Support-based: T1 0.95, T2 0.96, T3 0.95
Intentions to leave	Intentions to leave current job and nursing profession (Kim et al.,)	3 (one item replaced to capture longer-term intentions)	Range 1–5 Low intentions (score<2.5), moderate intentions (score 2.5–3.5), strong intentions (score>3.5)	Cronbach alphas: T1 0.81, T2 0.81, T3 0.88

Both the interview schedule and survey were developed in collaboration with our Staff Advisory Group.

### 2.4 Ethical approval

We received ethical approval from the University of Leeds Research Ethics Committee (AREA 20–041; 3/12/20) and the Health Research Authority (IRAS Project ID: 290,616; 12/11/20).

### 2.5 Data analysis

Audio files were transcribed, and familiarisation took place by listening and re-reading transcripts. To understand how nurses made sense of their experiences across time, pen portraits (Sheard and Marsh, 2019) were created. Initially two authors (AD & HH) created pen portraits for three participants across each Trust, using a thematic analysis approach to identify key factors influencing nurses’ sensemaking of their redeployment experience, and met to discuss theme concepts. The aims at this first stage were to understand and define the core focus of the analysis. Here, identity was highlighted as a concept that changed for nurses during their redeployment. In the second stage of analysis a basic framework was designed to support the analysis of each participants data and the development of their pen portrait. This included nurse and organisation identity shifts across time and contributing factors for example: nurse specialty, personality and team dynamics. An inductive, iterative approach to this was taken, with the pen portrait framework being refined to meet the needs of the data. During stage three, two authors (AD & RSE) used deductive thematic analysis to develop pen portraits for each participant. These outlined the key factors from the interviews that nurses described contributing to their experience, their sensemaking process and consequences of their redeployment. Whilst creating the pen portraits, several meetings were held between the authors (AD, HH, RSE & KU), supporting an iterative approach, and enabling refinement. Following these meetings summaries of participants’ journeys were added to the pen portraits. Once most of the pen portraits were completed, these meetings were used for the fourth interpretation stage of the data to establish any clear patterns or groupings within the pen portrait summaries and their corresponding survey data (quantitative perceptions). Three distinct ‘journeys’ were identified representing how nurses differentially interpreted their redeployment experience. From this, an analysis framework was developed and refined to outline, within each ‘journey’, the factors contributing to changes across time. Two authors (AD & RSE) developed pen portraits for a portion of the same participants to ensure consistency and reliability across authors in developing the pen portraits and assigning ‘journeys’. The authors’ developed conceptually similar pen portraits for these participants and assigned the same ‘journey’ type. Should disagreements have arisen the authors had conversations to discuss these.

We looked at common factors associated with each journey and how, across the three surveys, each group reported their burnout (both disengagement and exhaustion), psychological distress, turnover intentions, and performance (both task-based and support-based) (see Table 1). Scales that have been validated previously in the nursing context were used and although no information is available about their validity during a pandemic, the scales were reliable across the time points in this sample.

## 3 Results

Sixty-two nurses with different redeployment experiences (i.e. low to high risk, high to low risk, shielding) were recruited at T1 (see Table 2). Of these, 53 (85.5 %) were interviewed again at T3 and 38 (61.3 %) completed questionnaires at all time-points (15 completed surveys at two time-points, eight completed one and one did not complete any). Participants had a wide range of work experience from 2 years to 38 years qualified (mean 16.68 years; median 17 years; standard deviation 10.05 years).

**Table 2:** **Participant characteristics**.

Characteristic		Number, Percentage
Gender	*Male*	6, 9.6%
	*Female*	52, 83.8%
	*Prefer not to self-identify*	1, 1.6%
	*Missing*	3, 4.8%
Ethnicity	*White*	49, 79.0%
	*Asian*	5, 8.1%
	*Black*	1, 1.6%
	*Mixed*	2, 3.2%
	*Other*	2, 3.2%
	*Missing*	3, 4.8%
Trust	*A*	25, 40.3%
	*B*	21, 33.9%
	*C*	16, 25.8%
NHS pay band	*5*	17, 27.4%
	*6*	22, 35.4%
	*7*	19, 30.6%
	*8*	1, 1.6%
	*Missing*	3, 4.8%
Nurse training	*In the UK*	49, 79.0%
	*Outside the UK*	10, 16.1%

**Fig. 1:**
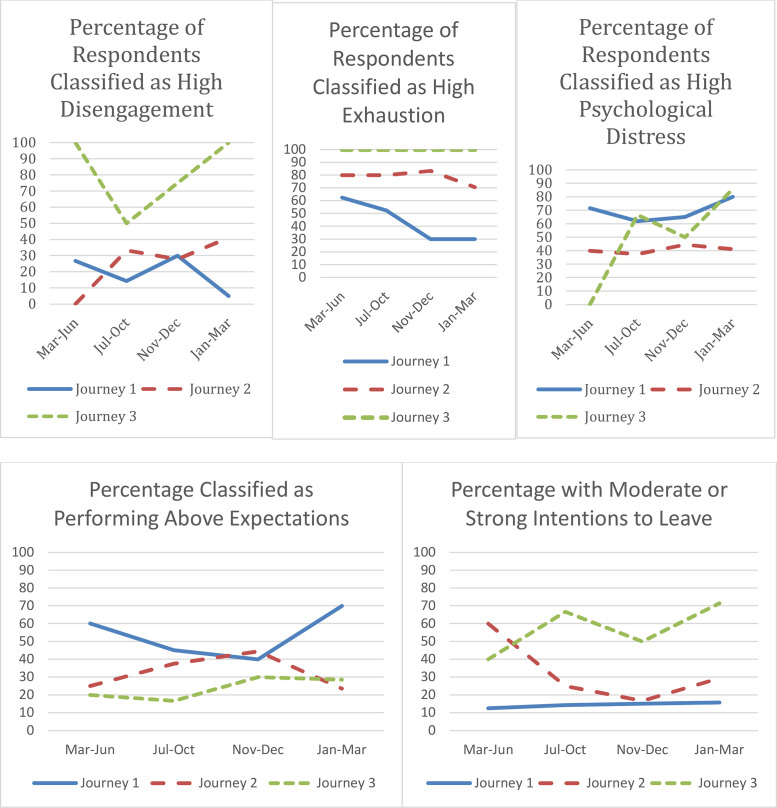
Burnout (Disengagement & Exhaustion), Psychological distress, performance and turnover intentions over time *(As the three data points were collected across overlapping timeframes, here the data is collated across time rather than data collection points)*.

**Fig. 2:**
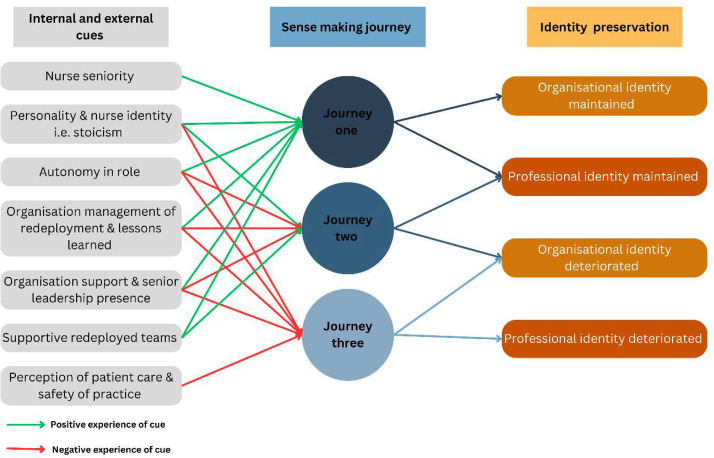
The internal and external cues that influenced the nurses sense making journeys and professional and organisational identities.

For all nurses, working through the protracted state of flux and dramatically changed work circumstances had effects on their psychological distress and burnout: all reported evidence of distress (which, for 55 %, was ‘severe’ in their first response) and 89.3 % were classified as having moderate (49.3 %) to high (40.4 %) burnout. However, our pen portrait analyses revealed key differences in the way nurses interpreted their redeployment experiences and, accordingly, the impact on well-being and work outcomes differed. We found that the way in which nurses made sense of their experience was influenced by, and in turn influenced, how they perceived themselves as professionals and as members of their particular organisation. Three different sense-making ‘journeys’ relating to identity were apparent (see Fig. 1). In journey one, nurses (n = 28) maintained both their professional nurse identity and organisational identification. Those in journey two (n = 24) maintained their professional identity, but their organisational identification deteriorated. In journey three (n = 10), nurses’ professional identity and organisational identification both deteriorated. These three journeys are now described in detail, outlining the cues that influenced sense making (see Fig. 2) and consequences of redeployment for nurse well-being (burnout and psychological distress), performance, and retention in each case.

### 3.1 Journey one: Organisational identification and professional identity maintained

The first nurse group we identified managed to make sense of redeployment in a way that did not undermine their organisational identification or professional identity. These nurses’ identities were maintained and stable throughout the study. For example, one nurse said, “*I do still love my job despite the pandemic” (2B29)* and another said, “*I just kind of believe more in the core values of the [organisation]” (2O29)*.

Some nurses in this group initially reported feeling anxious (e.g., “*you do feel a bit apprehensive because you gotta go to somewhere where you're out of your comfort zone with other staff members who you don't know”, 2B13)* but most of the emotion-focused coping these nurses engaged in related to patient acuity and the distressing way that patients were dying, more so than redeployment specific events. These coping strategies included, organisation support, taking time away from work to spend time with friends or family. As can been seen in Fig. 1. these nurses reported moderate levels of distress and burnout throughout the research period and the narratives suggest this was attributable to these experiences. For example, one nurse said, “*you couldn't have relatives then and we literally had a small mobile phone that we were doing Facetime to relatives on, and we were taking CPAPs off these people while the relative was dying and that was only connection they had. So, after June, I think after about three in one day, and that was when I said I can't do this anymore…The support was there, but I just needed to take it a little bit of time off” (2B11).*

This sensemaking journey was associated with several internal and external cues. Most of this group were band 6 nurses or higher (that is, relatively senior) and therefore are more likely to have a contextual understanding of their redeployment. They were more involved in decision making or had greater understanding of the justification and management of redeployment for the whole hospital in terms of staffing needs and patient safety. For example, one nurse said, “*I think a one to one with the manager would have been helpful like with the matrons but also, I know that they were very busy, mainly with the- the huge staffing shortage cause, […]. So, I think, pragmatically there probably wasn't any time in the day to be able to facilitate that but, it would have been nice from a wellbeing point of view to have that check […] but yeah logistically I don't think it would have been possible.”* (2L4)

Perhaps because of this, nurses on this journey made sense of their mental health harm by attributing it to outside their organisation - to the wider COVID context, NHS, or government pressures – and thus minimising organisational blame. For example, one said “*you couldn't blame them, they did try with the first wave…everyone was struggling, the whole country, whole nations, whole world was struggling with it” (2O27)*. Attributing harm to external factors, rather than internal organisational decision making, appeared to protect both the nurses’ organisational identification and their perceived wellbeing and performance.

One exception was a newly qualified nurse, whose junior status was used as a cue for a “learning” interpretation of redeployment. They said “*I don't really have any negative feelings towards being redeployed and it was obviously my choice to be as well, I don't know if I said that before. Like I learnt so much, I worked with people that are way above my pay grade and like skillset, so that was amazing, and I still think about the things that I saw and the things I was exposed to and refer back to them in like a professional way” (2L29).* However, what is notable in this instance is that redeployment was their “choice”.

This perception of choice and agency was apparent in the sensemaking of other nurses in this group. Most purposively sought their own redeployment arrangements or volunteered when the redeployment request was made, e.g., “*There was another email to say do people want to go for a short period of time to ITU and I volunteered because I was actually [thinking], ‘It might help me feel a little bit more competent in this situation’” (2L14).* Thus, these nurses engaged not only in emotion-focused coping but also problem-focused coping: “*I'm someone who likes to know what's happening and likes to be in control. And so, I was almost trying to kind of pre-empt and have the information up front so that I wasn't suddenly put in this position where I felt like, ‘Oh my goodness, I don't know what's happening’” (2O2).*

However, it was recognised that this problem-focused coping was enabled only due to their situation: these nurses were typically more senior, worked in a leadership role or received redeployed nurses. For example, one said, *“appreciate I have the choice to go, and I made those choices and I also have the choice to come back earlier and I chose to stay, but I know like a lot of people didn't have choices…I think if I'd have been moved with no choice it would have been even harder personally*” (2L10). Having this autonomy and control within redeployment (i.e., volunteering) enabled these nurses to be redeployed to a role matching their existing skills, allowing them to work within their competencies and manage stressors. Indeed, developing their skillset enhanced their nursing identity, e.g., “*So I feel more of a nurse. I've explained that one already. I think it increased my own confidence. Because by describing myself as the theatre nurse, and I'm not a proper nurse, was probably saying that I had some fears of gaps in my knowledge since training had finished” (2B14).*

Most of these nurses also described feeling supported and appreciated by their peers, line manager, and organisational leadership enabling emotion- focused coping, e.g., “*I think that personally what's already been given is enough, from a Trust point of view…I do think like the extra day of annual leave was lovely like, and I did feel valued after that because there was this. They sat up and they understood how much had been given during that time” (2L4).* Another said, *“I think they're listening. It's good to know that they're listening, and they're not just saying well don't matter” (2B11).*

Similarly, following the pandemic ‘peaks’, these nurses described how their organisation's leaders had learned lessons and were flexible in their responses to the pressure: “*I think wave 3, from my perspective I feel they've got it now. Like everything feels calmer. Wave 2, I felt like we were skating a little bit on thin ice at times… But I spoke about better support systems and all that sort of thing and, I'd like to think they listened to some of it [chuckles] … because after that, every shift I worked on the COVID adult ward I had someone I knew” (2L4).* This contributed to nurses’ perceived sustained identification with their organisation, as they believed their organisation was doing all it could in the context of the external pressures.

At the same time, all nurses within this group believed that their personality characteristics were crucial to their redeployment experience. Stoicism was considered central to ‘being a nurse’, enabling them to appraise their experience as an opportunity and to learn from the positives. For example, one nurse said “*I've chosen to be a nurse. I've chosen this career, so and it's not hunky-dory all the time, it's not all, you know, roses that. It never will be because it's the job*” (2L4). Thus, their professional identity was being used to make sense of the situation and promote emotion-focused coping.

This was further demonstrated in time-point three as these nurses discussed actively seeking and only reflecting on the positives of their experiences. For example, one said, “*I've tried to look back on it all as just a big learning curve really, It taught me a lot about myself, it taught me that I'm quite resilient and I just crack on and get it done really, so I've tried to look back on it in more of a positive way really because it's essentially been 2 years of my life and you don't want it all to be negative*” (2B57).

### 3.2 Journey two: Devaluation of organisational identification but maintenance of professional identity

The second journey of redeployment sensemaking we identified involved maintaining their sense of professional nurse identity, but identification with their organisation was undermined over time. For example, in their first interview one nurse said “*I guess I can see now… the fact that I did still know how to nurse, I did still know how to relate to patients. I could still do the job”,* but in the next interview they also said, *“And I knew we were in an emergency situation and staff had to be placed where they were needed and what have you, but the Trust have very strong principles around valuing staff and respecting staff, and I felt that just went out of the window*” (2O17).

The survey data, reflected in Fig. 1, showed that these nurses had significant health concerns and that their health and performance shifted throughout the COVID-19 redeployment more than those who maintained both identities. In particular, this group felt they were performing above expectations during the period over June-August 2021 (following the end of a national lockdown) with concurrent increases in turnover intentions, disengagement and exhaustion, e.g., “*So now we expect to do far more than we can do” (2B21)* and “*I'm quite a strong person, but it does play with your mental health when you're in a situation where you don't really want to be there and you're worried about what is going on and having the correct support around you” (2B20)*.

Compared to journey one, the external cues used in sensemaking were harmful in this group particularly with regards to organisational identification. These nurses felt disempowered and had little control or voice e.g., “*it wasn't managed well at all. You know you didn't have a choice. I didn't have a choice whether I went into ICU and in terms of managing people and getting the best out of people, that's not what you do [it] But they did not prepare us in any way to deal with what we dealt with when we first went into ICU. Absolutely didn't” (2B20).*

These nurses were also more likely to be redeployed to a ‘high risk’ area and described how their redeployed role did not match with their existing skills. This competency-gap contributed to them feeling like an object, undervalued by their organisation, e.g., *“Before they used to care about you, you know, like you're a person emotionally and professionally what you're doing…now it's just like numbers…They don't care. They see that there there's not enough people and we are struggling, but they don't care” (2B21).*

In contrast to Journey 1, harms were attributed to actions (or inaction) by the organisation rather than the external crisis; specifically, how the redeployment process was managed. Nurses perceived there to be poor management and communication, insufficient preparation for redeployment, inadequate support, and senior leader visibility in Trusts. For example, one said “*there were three nurses from my department who were kind of forced out, edged out almost… no, not almost, that was exactly what happened…I was told that the very next day I'd be going to the COVID ward with no kind of heads up I thought it was really unfair” (2B42).* Furthermore, the view that the organisations failed to learn from and plan for redeployment in subsequent waves and post-pandemic management eroded their trust, e.g., “I'm pretty p***ed off about it really. It was just so disorganised, a complete knee jerk reaction” (2B50).

A sense of injustice pervades this journey narrative. When describing their redeployment experiences, fairness was a key concern for most nurses, who regarded the redeployment selection process as unfair or the decision process as lacking transparency, e.g., “*As a nurse you put your patients first and, and that's what's important isn't it but, you know there are other people that haven't been or being asked to do anything over and above their current role. And to be fair and equitable, I think that should be the case, things should be done fairly, and it shouldn't be disproportionate” (2B20)*. Similarly, “*Initially it did feel like I was being targeted, you know, being a junior member of staff” (2B42).*

However, whilst these nurses experienced a decline in organisational identification, they nevertheless maintained their professional identity. Many in this group viewed redeployment overall as not a ‘bad experience’ as it contributed to this identity, e.g., “*it was a good experience just to say you know what, you can do this. You can do this and go elsewhere and do other things and do some good” (2O16).* This may also be helped by having supportive teams during their redeployment period providing opportunities for emotion-focused coping. For example, one nurse said, “*The team were very supportive, and I think I was very lucky when I got moved because that was at the beginning of the pandemic […] so there were a lot of hands on deck” (2B42).*

Importantly, like Journey 1, maintaining one's professional identity influenced how these nurses made sense of their redeployment experiences: redeployment was seen as intrinsic to being a nurse. For example, one nurse said “*I would just do it. That's what nurses do, and I guess that's what nurses will always do. That's what I would do” (2O16).* Thus, because nurses were resilient and stoic, they would be too: despite typically working outside comfort zones, these nurses felt they did their duty, e.g., *“As a nurse, as a professional, I played my part and stepped forward and did what I needed to do, and that makes me feel good” (2B20)* and *“I would just do it. That's what nurses do, and I guess that's what nurses will always do…it isn't really a choice, but we just knuckle down and get on with it.” (2O16)*. Thus, they were using their professional identity as a lens through which to make sense of the situation and determine the appropriate response.

### 3.3 Journey three: Devaluation of both organisational identification and professional identity

The last sensemaking journey we identified resulted in an undermining of both the nurse's organisational identification and professional identity. For example, one nurse said, *“nursing, it was all I ever wanted to do, and I got into nursing and I could accept that you were busy and short-staffed and people whinging about pay and things like that, I sort of accepted all that, but when you are […] stretched too far, pushed too far it's put me off nursing really […] my opinion of nursing's not very good at the moment.”* The same nurse also described their lack of faith in their organisation, “*I don't have a lot of trust… so if I was to be redeployed again and they said those words ‘you're going to support a ward’, I know it's not the truth. […] because I had been moved to support a ward, but more often than not it was me and my other colleagues […] keeping the ward afloat. So, it wasn't support, we were definitely in the numbers […] I'd be, it's just a lack of trust I think that I feel is a negative part.” (2B56).*

As can be seen in Fig. 1, nurses undergoing this sensemaking journey described the worst mental health experiences within our sample. The interviews showed that many experienced long-term consequences, including grief, e.g., *“I am now [laughs] you know, on the cusp of being burnt out and really struggling, and getting help around that, which is great, but it has, it has almost broken me professionally and has made me erm… very sad” (2L12).* These nurses felt anxious and worried about what they perceived was an endless and unsafe situation: “*I've just never, ever seen it so short on staff and the numbers that they're having to leave wards on is just unsafe and it's scary and it just doesn't feel like there's going to be any change any time soon” (*2B18).

Moreover, the nurses struggled considerably with the quality of care that they were able to deliver during the pandemic: the shift to task-based nursing challenged their values and prevented them from ensuring the overall patient experience. In common with Journey 2 nurses, this nurse group felt their task-based performance exceeded expectations from June-August 2021 with a concurrent increase in psychological distress; however, unlike those in Journey 2, these nurses experienced enduring negative outcomes including high levels of turnover intentions and psychological distress after this period. One said, “*I don't know what else you can sort of physically do to make sure, you know, that everything is done. Because, as horrible as it sounds, you kind of are at the point at the end of a shift is, do you know what, that bit of paperwork's not done, but my patient's comfortable, settled, breathing” (2B18).* Nurses described losing the joy from nursing and feeling that the role was no longer patient experience focussed. Another said, “*not particularly nice thing to admit but my patience for patients is very low and …it's difficult to, you know, difficult to summon the empathy and the sympathy that used to come very easily. I wouldn't necessarily say, I wouldn't necessarily be as quick to admit that my care is worse, just I find it harder to deliver or deliver in the same kind of nice way” (2L21).*

Their diminished ability to deliver care during redeployment, coupled with ongoing mental health struggles also undermined their identity as a nurse: “*it's dread of going to work and it's awful because, you know, patients still need you and everything…but I just don't feel that you think you can do as well as what you could have done previously” (2B18)*. As a result, these nurses were also more likely to have moved roles or be applying for new posts. One nurse commented that, “*There was like a palpable bitterness, but I think no one ever spoke up about it. Some people quit. Some people left, and that's the main reason […] I wanted to move” (2L2)*.

As with the other journeys, both internal and external cues were involved throughout this sensemaking process. Interestingly, the internal cues differed for this journey. Unlike the other two, nurses in this group did not use (or have available to use) the nursing identity that triggered stoicism and resilient behaviour. On the other hand, unlike those in the other sensemaking journeys, these nurses often referred to negative experiences of redeployment before the pandemic, e.g., *“So even before the pandemic if a matron came round it was…you need to move elsewhere, we need to cover this…I know there's pressure for them and, I know they're just doing their role but, when it feels like it targets you personally it's not nice” 2B56*. Thus, they may have had a pre-existing schema of redeployment which was used to make sense of the pandemic redeployment.

Nonetheless, echoing the experience of journey two nurses, but contrasting with those in Journey 1, this nurse group felt disempowered through redeployment, e.g., *“I've never felt more of a number than I felt on that day, and I've never felt so disheartened working for the trust as I have felt this past 12 months” (2B4)*. This was exacerbated by feeling that they had lost their voice: “*you do feel that you are a little bit powerless, it doesn't matter how much we shout and stamp our feet and try and put our point across, it's still not necessarily, you know, I think people hear what we say but they can't actually do anything about it” (2O12).* Another said, “*You're just plunged into the deep with no orientation and no follow up asking how you are and stuff like that. And at the beginning, people were voicing their concerns and then they were just kind of ignored or like told to just deal with it and stuff so that was a big factor” (2L2).* These findings suggest that these nurses felt discouraged from engaging in problem-focused coping; their learned helplessness (Abramson and Seligman, 1978) meant that they could cope only by managing their emotions.

This emotion-focused coping, however, proved difficult for those in this sensemaking journey as all but one nurse in this group were redeployed to a ‘high risk’ area; the other was a critical care nurse who received redeployed nurses. Like Journey 2, these nurses felt that their redeployment role matched neither their skills nor competencies, resulting in some of them feeling de-skilled; contributing to professional identity erosion: *“when it came to the qualified roles and responsibilities, they were quite daunting in those first few shifts, cause I've not done it for years…so, it was hard to adapt…I did feel out my comfort zone with a lot of things” (2B56).* Moreover, they experienced poor communication, management, inadequate preparation, poor senior leadership presence and no support reducing the opportunity for emotion-focused coping. For example, during our first interview with them, one nurse said “*The trauma I would say is obviously not something I'd want to do again, but I think a bigger factor is the way that things were handled. Because you know it's kind of frustrating when you work in a caring profession and then instead of like caring, as you know the mission and vision of the whole trust is ‘we care’, but then instead of prioritising that, it's actually politics that's way up there, and everything else is just like woke, you know, tokenism” (2L2).* By the third time point, this nurse was more frustrated because they felt their organisation had not learned from the pandemic or listened to the frontline nursing staff: “*after that first wave there was, there were a lot of, doctors and nurses who got together and, discussed and said look what happened was really terrible, how they handled the staff was really terrible they shouldn't happen again and then they, they started some programmes and then they made clear some policies and protocols like, you know empowering staff to voice out. But then there was the second wave and essentially it was the same thing, just on a smaller scale…I feel like the top management, are quite disconnected, and I feel like they should listen more” (2L2).*

## 4 Discussion

Our longitudinal multi-method study demonstrated the sensemaking processes and cues that led to three different experiences of nurse redeployment and perceptions of psychological distress, burnout, turnover and performance.

We found identity was at the heart of differentiating the emotion-focused coping narratives. Identity is central to sensemaking theory: It has been outlined as the first of seven properties of sense-making (Maitlis and Christianson, 2014) and research has shown that one's identity affects how one makes sense of the situation (e.g., Gioia and Thomas, 1996). Moreover, because identity is a long-term goal (see e.g., Cropanzano et al., 1993; Unsworth, Yeo and Beck, 2014) it is likely to be even more crucial in understanding long-term extreme events. Indeed, we found that maintaining their nursing identity cued traits of stoicism and resilience in Journeys 1 and 2 which allowed nurses to continue performing their duties throughout the COVID-19 redeployment.

However, because our research was conducted longitudinally across the long-term extreme event, we were able to move beyond this static assessment and uncover a feedback loop demonstrating that one's sensemaking affected one's identity. The cues that the nurses were exposed to prompted different patterns of sensemaking which led to either the maintenance or the devaluation of their organisational identification and professional identity. Less work has examined how sensemaking plays a role in determining one's identity (Brown, 2015), however, there is evidence that people use sensemaking to not only understand their situation but also who they are and what they are doing (e.g., Vough et al., 2020). Indeed, following trauma, sensemaking may be crucial to allowing a person to determine their sense of who they are Maitlis (2009). Our research extends existing knowledge by outlining cues that differentiated three sensemaking journeys leading to different identity outcomes: maintenance of both, devaluation of one, or devaluation of both professional identity and organisational identification.

We can also see the circularity of identity and sensemaking when comparing journeys 1 and 2 with journey 3. Nurses with a devalued professional identity were no longer able to draw on the “nurse” characteristics of stoicism and resilience. These internal cues were crucial to those in journeys 1 and 2, enabling them to put on their “nurse hat” and “carry on with the job”. Thus, identity affected sensemaking (e.g., I'm a nurse therefore I see this as a situation which I can and have to deal with) but because sensemaking affected identity (e.g., this situation is too overwhelming for me to engage in nursing) that protective factor was no longer available. This means that there are positive and negative reinforcement cycles that can emerge from sensemaking, with negative cycles resulting in potentially serious harm. We know that emotions and resources can operate through gain and loss cycles (e.g., Fredrickson, 2004; Hobfoll, 1989) and our research suggests that sensemaking may follow a similar process.

Nonetheless, recent research suggests that the seminal sensemaking approach may be too human-centred, leading to the implicit assessment that people could have better outcomes if only they had engaged in better sensemaking Jensen and Mahmud (2023). This is particularly relevant to this study as, although sensemaking journeys were associated with the outcomes, we also found that differences in the external environment played a substantial role in which sensemaking journeys were undertaken. Those nurses able to place their individual narrative within the broader organisational context, who had more support and/or more control (i.e., Journey 1) were more likely to have “effective” sensemaking, while those nurses who did not have this support or control were not. Thus, we contribute to the sensemaking literature by highlighting the different patterns of sensemaking that emerge when there are different external cues available.

Three aspects of the environment appeared to differentiate the sensemaking journeys: control, support, and contextual awareness. First, having a sense of agency and control during COVID-19 redeployment appeared crucial in the sensemaking narratives: those with control (Journey 1) were better able to manage their emotions and had better outcomes than those who did not (Journeys 2 and 3). It could be that insufficient control during a crisis resulted in sensebreaking (Pratt, 2000) and nurses in the latter journeys were left “lost” in a state of dissatisfaction trying to seek resolution. Previous literature has also highlighted the need for autonomy to facilitate a positive nurse redeployment experience in the pandemic Kissel et al. (2023); Maben et al. (2022). *Thus, a clear practical implication of this research is the need to instigate a system of redeployment that provides a level of control and choice to the individual nurse to ensure that the redeployed role match their skills and competencies, even during periods of mass redeployment.* Rapid implementation of this may be challenging during a crisis setting, therefore we recommend that such systems are developed and used during usual service delivery and scaled up in response to a crisis or surge. Applying a systems approach during normal service delivery may also support preparedness of organisation and nurses in preparation for future crisis settings or surges minimising the chances that nurses, such as those in our Journey 3, embed negative schemas of redeployment based on pre-pandemic redeployment experiences.

Second, the support perceived by the nurses differed across all three journeys with less support associated with more identity devaluation. Although we are not aware of research that links organisational support with identity or identification, there is widespread recognition that such support creates a stronger relationship, which then allows for deviations from a strictly reciprocal contractual arrangement without negative effects Blau (1964); Cropanzano, et al. (2017); Cropanzano and Mitchell (2005). Furthermore, the lack of support in redeployment has similarly been associated with negative impacts on nurse wellbeing and retention during COVID (Ballantyne and Achour, 2023; Kennedy et al., 2022; Vera San Juan et al., 2022). *Thus, we recommend that nurse managers facing an ongoing extreme event recognise the need for as much direct support as possible from the receiving team and line managers, this might also include setting up peer support groups or accessible counselling services.* We appreciate that managers find themselves in a difficult situation during extreme events and this might lead them to think in terms of “staff” rather than “people” Stapleton and Opipari (2020). Our findings show that this lack of support and “people” orientated management led to a devaluation of organisational identification and, ultimately, the professional identity. Any resources that are available to be used to help nurses reduce their stressors and managers to manage redeployment should be deployed.

Finally, it appears that an attribution that the organisation could have better managed the process led to a devaluation of the nurses’ identification with the organisation (Journeys 2 and 3). Yet when nurses attributed the management problems to the crisis itself, rather than to the organisation, or that their organisation had managed well despite the external crisis, their identification was maintained. Interestingly, the group who maintained their organisational identification were more aware of the ways in which senior managers were themselves struggling with the pandemic. It could be that more transparency around the conflicts and ambiguities that leaders are facing during long-term extreme events might allow all employees (and not just those with seniority or insider-access) to incorporate this into their sensemaking and maintain their identification. *Thus, we recommend that those tasked with managing nurses and other employees during a long-term extreme event are transparent in the problems faced by the organisation, fostering open channels of communication where nurses can express concerns and receive timely updates about decisions and the rationales. This would enable people to attribute the problems to the external situation or crisis rather than the management of that crisis.*

### 4.1 Limitations

Our research was initially designed to be conducted in three distinct stages, enabling a clear comparison across sites and across time points. Unfortunately, the nature of the pandemic required us to stagger our access across the hospitals to minimise burden leading to inconsistent data collection points across the Trusts. Ideally, future research into ongoing extreme events may be able to overcome this issue and investigate in more depth the effects of cues over time. Similarly, we had hoped to gather data from nurses from a range of ethnic backgrounds to understand how that affected the sensemaking process. Unfortunately, our efforts were only mildly successful, as most of our sample were UK trained, white, female nurses therefore limiting the generalisability across gender and ethnicity. Having a more diverse sample would have enabled us to better understand the experiences of nurses who had an increased risk of contracting COVID-19 and how this may have influenced their sense making of their redeployment.

## 5 Conclusion

In conclusion, our research uncovered the interwoven pattern of identity and sensemaking as nurses tried to cope with the long-term extreme event of COVID-19 redeployment. We extended existing knowledge by demonstrating, not only that identity affects one's sensemaking, but also that sensemaking led to the maintenance or devaluation of organisational identification and professional identity. Given the stark differences in how nurses perceived their psychological distress, burnout, turnover intentions and performance across these patterns, their importance is apparent. Our identification of external cues: control, support, and contextual awareness, that affected sensemaking provides clear guidance for those managing people who might find themselves facing a long-term extreme event. This study and the wider research project supported the development of national recommendations for NHS nurse redeployment management and a best practice guide (these can be accessed at: https://yqsr.org/redeployment-of-nurses-in-hospitals-redeploy/).

## Funding sources

This is independent research funded by the National Institute for Health and Care Research (NIHR) (Health and Social Care Delivery Research, Lessons from the frontline: The impact of redeployment during Covid-19 on nurse well-being, performance and retention, NIHR132041) and carried out at the NIHR Yorkshire & Humber Patient Safety Translational Research Centre (PSTRC). The views expressed in this publication are those of the author(s) and not necessarily those of the NIHR or the Department of Health and Social Care.

## CRediT authorship contribution statement

**Alice Dunning:** Writing – original draft, Visualization, Validation, Project administration, Investigation, Formal analysis, Data curation, Conceptualization. **Hannah Hartley:** Writing – review & editing, Project administration, Investigation, Formal analysis, Data curation, Conceptualization. **Kerrie Unsworth:** Writing – original draft, Supervision, Funding acquisition, Data curation, Conceptualization. **Ruth Simms-Ellis:** Writing – review & editing, Supervision, Methodology, Formal analysis, Data curation, Conceptualization. **Michael Dunn:** Writing – review & editing, Supervision, Funding acquisition, Conceptualization. **Angela Grange:** Writing – review & editing, Supervision, Funding acquisition, Conceptualization. **Jenni Murray:** Writing – review & editing, Supervision, Funding acquisition, Conceptualization. **Jayne Marran:** Writing – review & editing, Validation, Supervision, Conceptualization. **Rebecca Lawton:** Writing – review & editing, Supervision, Methodology, Funding acquisition, Conceptualization.

## Declaration of competing interest

The authors declare that they have no known competing financial interests or personal relationships that could have appeared to influence the work reported in this paper.
